# Violence against Women Living with HIV: A Cross Sectional Study in Nepal

**DOI:** 10.5539/gjhs.v4n3p117

**Published:** 2012-05-01

**Authors:** Nirmal Aryal, Pramod R. Regmi, Naba Raj Mudwari

**Affiliations:** 1 Department of Health, Physical and Population Education, Central Campus Kirtipur, Tribhuvan University, Kathmandu, Nepal; 2 Section of Population Health, University of Aberdeen, Medical School, Scotland, UK

**Keywords:** Violence against Women, HIV, AIDS, Nepal

## Abstract

**Background:**

Violence against Women (VAW) and Human Immunodeficiency Virus (HIV) both constitute major public health issues and there is an increasing evidence of their intersection. Data are sparse on the intersection of VAW and HIV in South Asia region. We aimed to identify different forms and magnitude of violence incurred by women living with HIV, and analyse causes and consequences.

**Methods:**

A cross-sectional study was conducted among 43 HIV positive women in three districts of Nepal, in the period of March-May 2008. Data was collected through semi-structured interview questionnaire.

**Results:**

The vast majority of the participants (93.02%) had suffered from at least one form of the violence. The prevalence of violence rose up sharply after being diagnosed with HIV positive than before (93.02% vs.53.5%). Forty-five percent of the participants reported their husbands being main perpetrator of violence. Self-humiliation and health and treatment problem were the major consequences of violence as reported by 90% and 77.5% of the participants respectively.

**Conclusion:**

Violence was observed to be highly prevalent among women living with HIV in Nepal. Further larger and nationally representative researches are imperative to better understand the cross-section between VAW and HIV. Our finding recommends to prioritizing programs on social aspects of HIV such as violence.

## 1. Introduction

Violence against Women (VAW) and Human Immunodeficiency Virus (HIV) both constitute major public health issues and there is an increasing evidence of their intersection. Evidence also suggests that HIV positive women are highly vulnerable to the different forms of violence, predominantly physical, sexual and emotional violence (Hale & Vazquez, 2011). Violence on HIV positive women has also been recognised as a major barrier to obtain regular medical care for HIV related diseases (Lichtenstein, 2006).

A number of studies (Watts et al., 1997; Gielen et al., 2000; Fonck et al., 2005), but not all (Gruskin et al., 2002) have suggested higher prevalence of violence among HIV positive women and also significantly greater than HIV negative counterparts. For example, a systematic review carried out by Campbell and colleagues (2008) showed the prevalence of physical violence by intimate partner ranged from 60 to 67% and sexual violence in the range of 32 to 46%. This review globally included 71 original and peer-reviewed studies conducted between 1998-2007. Also, an earlier study in Tanzania investigated that the odds of reporting partner violence was 10 times more in younger HIV positive women (< 30 years) than the HIV negative women [OR = 9.9, 95% C.I. 2.67, 37.37] (Maman et al., 2002).

As of September 2011, 18,396 cases of HIV were officially reported in Nepal, however, total number of people living with HIV was estimated to be 55,626 and adult HIV prevalence was 0.33% (NCASC 2011). According to the United Nations General Assembly Special Session (UNGASS) country progress report 2010, more men than women contracted HIV in a ratio of 2.9:1 (UNAIDS 2010). Nepalese women are vulnerable to the risk of acquiring HIV mainly due to the risky sexual behaviour of migrant husband and sex trafficking compounded with gender-based discrimination (Sarkar et al., 2008; Silverman et al., 2007; Smith-Estelle & Gruskin, 2003).

There is a paucity of literatures on violence among HIV positive women and the vast majority of the studies were based on American and African setting. As far as we are aware, none of the studies were conducted in Nepal about this issue. In this study, we aimed to (a) identify different forms and magnitude of violence incurred by women living with HIV and AIDS, and (b) analyse causes and consequences.

## 2. Methods

This was a cross-sectional mixed method study conducted in Chitwan, Makwanpur and Parsa district of Nepal. These sites were selected because these are prominent HIV risk zone of the country which is hugely attributed to the migration and sex work in the highway districts. The data was collected between March-May 2008.

The study participants were selected through convenient sampling method as detail database of HIV women was not available for random selection and the study team had to rely on HIV related organisations of each district to gain the access of HIV positive women. Only those HIV positive women who were in regular touch of these organisations were approached individually by the study team. Forty three women living with HIV participated; 18 from Chitwan, 15 from Parsa and 10 from Makwanpur district (characteristics of the participants has been given in Table 1). According to the estimation of District AIDS Co-ordination Committee (DACC) in 2008, there were 114 HIV positive women in Chitwan district, 23 in Makwanpur district and 120 in Parsa district. The number of women who disclosed their serum status publicly was even lower, for example in Chitwan only 40 HIV positive women had disclosed their status during the study time. The number of participants in each district was based on access and those meeting inclusion criteria. Inclusion criteria were: clinical diagnosis of HIV positive, age 15 years or more and providing informed consent. Eleven HIV positive women denied to get involve in the study.

**Table 1:** Demographic characteristics of the participating HIV positive women

Variables	Frequency (N= 43)	Percentage (%)
*Age-group (years)*		
20-24	3	6.9
25-29	13	30.2
30-34	17	39.5
35 or above	10	23.2
*Ethnicity*		
Janajati	20	46.5
Brahmin	12	27.9
Chhetri	5	11.6
Dalit	6	13.9
Muslim	1	2.3
*Occupational status*		
Migrant worker / agriculture	21	48.8
Labour	13	30.2
Driver	5	11.6
Business	2	4.6
Social service	2	4.6
*Educational level*		
Literate	25	58.1
Illiterate	18	41.9
*Economic status*		
Poor	27	62.8
Medium	16	37.2
Well-off	0	0

A consent form was developed in Nepali and provided to the participants or alternatively it was recited for those participants who could not read. A written or oral consent was solicited. An ethical approval was granted from the Department of Health, Physical and Population Education, Tribhuvan University, Nepal.

### 2.1 Survey Procedures

Data was collected through semi-structured interview questionnaire which was developed in Nepali. Reliability of the questionnaire was ensured by piloting it among six HIV positive women of Chitwan district. The questionnaire was divided into two sections. First section consisted socio-demographic questions, whereas second section was directly related with research topic. Primarily, the questionnaire intended to seek information on prevalence of violence before and after HIV infection, types and magnitude of violence, perpetrators of violence and its consequences.

All of the data was collected by skilled female researcher with the assistance of HIV activists of each district as it is often argued that gender division is necessary to collect information on sensitive topics. Many authors have a notion that people would be more comfortable discussing the sensitive and complex matter with others of same gender (Ingham et al., 1999; Krueger & Casey, 2009; Regmi et al., 2011).

Mainly, the data was collected at the office of HIV related organisations, and few were collected at the place of convenience of the participants. Face validity was assessed with the consultations of relevant experts. Data was entered in Microsoft Office Excel 2007 and descriptive statistics was derived in frequency and percentage.

### 2.2 Definition of VAW

Definitions of VAW have become increasingly wider as it also depends on societal values and cultural norms, and these aspects vary across the countries. However, we defined ‘violence against women living with HIV and AIDS’ as proposed by Hale and Vazquez 2011 and modified it taking account of specific experience and contextual to Nepal. Particularly, we differentiated psychological and emotional violence (Box 1).

**Box 1**

**Physical Violence:** physical abuse and harm

**Psychological Violence:** verbal abuse, threat of abandonment/expulsion

**Emotional Violence:** isolation, discrimination, actions causing humiliation, shame and embarrassment

**Economical Violence:** restriction on access and use of financial resources, deprivation from basic needs

**Sexual Violence:** sexual abuse

## 3. Results

Table 1 shows that a total of 43 HIV positive women participated in this study. Of these, 18 (41.9%) were from Chitwan, 15 (34.9%) from Parsa and 10 (23.2%) from Makwanpur district. All participants were married, out of which 24 (55.9%) were widow. The vast majority of the participants (36 (83.7%)) contracted HIV positive virus by the husband. Sex trade was attributed for HIV positive in 7 (26.2%) participants. Twenty seven (62.8%) participants were of the age 30 years or more. Nearly half (20 (46.5%)) of the participating HIV positive women were from indigenous group (‘janajatis’ in Nepalese language). Among the participants, the higher proportion (27 (62.8%)) reported being financially poor.

### 3.1 Prevalence of Violence

Table 2 indicates that the vast majority of the participating HIV positive women (40 (93.02%)) were suffering from at least one form of the violence. The prevalence of violence rose up sharply as compared to before (23 (53.5%)) when the participants were free of HIV positive. Among the participants who were suffered from violence, the most dramatic increment in the prevalence, after HIV status, was observed in emotional form of violence (3(13.0%) vs. 31 (77.5%)). Interestingly, none of the participants had reported being subjected with economic violence before the HIV positive status, whereas, 16 (40%) of the participants reported so after being diagnosed with HIV.

**Table 2:** Prevalence of Violence before and after HIV Status

Variables	Before HIV status		After HIV status	
	Frequency(n=23)	Percentage (%) (95% CI)*	Frequency(n=40)	Percentage(%) (95% CI)*
*Violence Status (N=43)*				
With Violence	23	53.5 (38.9-67.5)	40	93.02 (80.7-98.3)
Without violence	20	46.5 (32.5-61.1)	3	6.97 (1.7-19.3)
Physical Violence	13	56.5 (36.8-74.4)	22	55.0 (39.8-69.3)
Psychological Violence	17	73.9 (53.2-87.7)	36	90.0 (76.4-96.6)
Emotional Violence	3	13.0 (3.7-32.9)	31	77.5 (62.3-87.9)
Economic Violence	-	-	16	40 (26.3-55.4)
Sexual Violence	1	4.3 (0.01-22.7)	-	-

* 95% CI = 95% Confidence Interval

**Figure 1:**
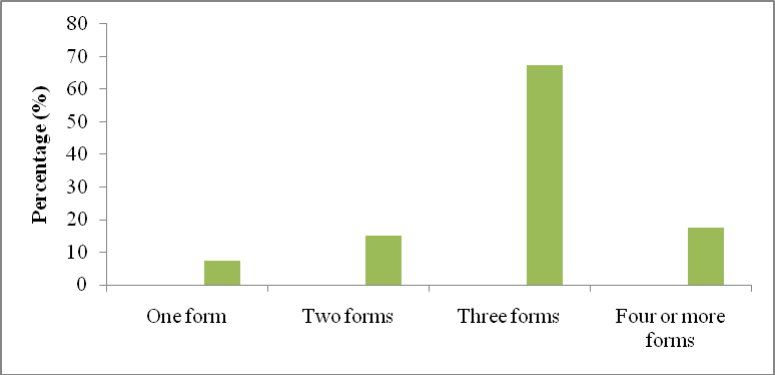
Number of forms of violence being perpetuated with HIV positive women

It was found from this study that the majority of the participats (27 (67.5%)) were concurrently afflicted from three forms of violence (Figure 1).

Among those subjected with physical violence, beating/slapping/kicking was reported by all (22 (100%)). It was followed by strangling (16 (72.7%)), stabbing (9 (40.9%)) and hit by firewood, utensils or broomsticks (5(22.7%)). For psychological violence, verbal abuse was the most common, as reported by 18 (50%) HIV positive women, which was followed by threat of expulsion (11 (30.5%). Likewise, among the participants afflicted from emotional violence, the higher proportion (12 (38.7%)) reported being isolated by the society members.

### 3.2 Perpetrators and Consequences of Violence

Table 3 shows that the husbands were the main perpetrators of violence as reported by 18 (45%) HIV positive women. It was closely followed by mother-in-law (17 (42.5%)). Maternal relatives were also found to be key perpetrators (14 (35%)

**Table 3:** Perpetrators of violence against participating HIV positive women

Perpetrators	Frequency (N=40)	Percentage (%)
Husband	18	45
Mother in law	17	42.5
Maternal relatives	14	35
Brothers in law	10	25
Father in law	9	22.5
Sisters in law	8	20
Son/daughter in law	2	5

**Figure 2:**
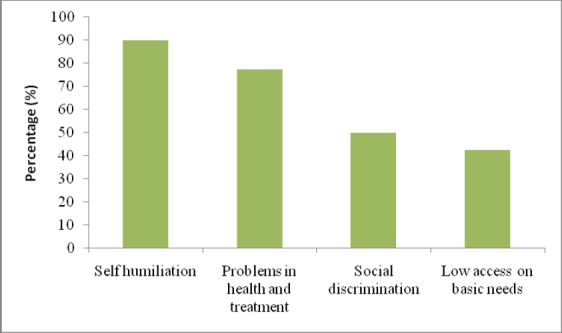
Consequences of violence reported by participating HIV positive women

Among the violence afflicted HIV positive women, self humiliation was the major consequence of violence, and was found in the vast majority (36 (90%)) of the participants (Figure 2). Likewise, health and treatment problem was observed to be the second-most (31 (77.5%)) key consequence of violence. Our study shows that one-fourth of the violence afflicted participating HIV positive women (10 (25%)) had formally lodged complain against the violence incurred upon them. This trend was more encouraging in Chitwan district where 7 (43.7%) participants filed complain, in sharp contrast to Parsa (2 (14.3%)) and Makwanpur (1 (10%)) districts. Most of the complaints were made in non-governmental organisations (NGOs) (6 (54.5%)), as compared to community / village groups (3 (27.3%)) and government agencies (1 (18.2%)). Notably, 8 (80%) of the complaining HIV positive women reported that the incidents of violence were reduced or stopped aftermath of complain.

## 4. Discussion

In the present study, we have demonstrated that the vast majority of the HIV positive women were suffering from at least one form of the violence, and the prevalence of violence increased by greater proportion after being diagnosed with HIV positive. Likewise, we also found that more than two-thirds of the HIV positive women were concurrently suffering from at least three forms of violence. The prevalence of violence presented in this study was higher than the most of other related studies. In our study, 93.02% of the participating HIV positive women reported being tortured with at least one form of violence. The findings from Zimbabwean study among 1000 participants were close to our findings where 84% of HIV positive women subjected with one or more forms of violence (Watts et al., 1997). In Ugandan study, 29.3% of HIV positive women reported any form of violence in preceding 12 months (Osinde et al., 2011). This study has examined only physical, sexual and psychological violence. Likewise, in another study among pregnant Nigerian HIV positive women, prevalence of any form of violence by intimate partner was reported to be 32.5%, and the scope was limited to physical and psychological violence (Ezeanochie et al., 2011). In contrast to other studies, a prospective cohort study among 1087 American women suggested that incidence of violence is lower in HIV positive women than negative ones (HIV positive 4.87 per 100 person-years, HIV negative 6.44 per 100 person-years) (Gruskin et al., 2002).

There are some methodological issues which may have contributed for higher prevalence of violence in this study. Firstly, we have included broad range of violence, including physical, economic, psychological, sexual and emotional ones. Most of the related studies have examined fewer forms of violence. We were cautious about the overlapping of the forms of violence and hence our questionnaire delineated specific examples for particular forms of violence (for e.g., in psychological violence we provided examples of verbal abuse, threat of abandonment and expulsion etc). Secondly, unlike many studies which only reported violence by intimate partner, our study included violence being perpetrated by anyone. Lastly, we examined violence afflicted before and after the HIV positive status, whereas most of the studies reported violence during the specific duration of time.

In consistent with other literatures, our finding has also supported that HIV status *per se* is the strong predictor of violence among women. In the present study, 39.52% of the participating women were induced violence after being diagnosed with HIV positive, whereas in an American study it was 45% (Gielen et al., 2000) and the Kenyan study investigated that HIV positive women were 80% more likely to suffer from partner violence during the life time period (Fonck et al., 2005).

This study has demonstrated that husbands were the main perpetrators of violence which accounts for 45% overall and 94.7% among those whose husbands were alive [24 (55.8%) were widowed]. This shocking finding suggests that almost all Nepalese HIV positive women were victimized by their own husband. Our finding corresponds with recent study on 1569 violence afflicted Nepalese women where 74% of the participants reported husband as the perpetrator (Women’s Rehabilitation Centre Nepal, 2011). Nepalese women, in general, have little autonomy and they are fully dependent on husband, even for the basic needs which raises their susceptibility towards the violence. Violence against women is often considered as a customary practice and has gained social acceptance as well. This tendency may have further aggravated in the case of participating HIV positive women. In the case of widowed participants, mother-in-law and other closely related in-laws subjected violence upon them. In some Asian countries, wives are often blamed for the HIV positive status of husband and his subsequent death, and evicted from the home. In similar setting of India, 91% of HIV positive women were blamed for their husband death and finally ejected from home (Development Connections, 2010). Thi and colleagues reported that in Vietnam, it was common that in-laws forcefully drove HIV positive women off from the house or they were separated from children (Thi et al., 2008)

Our study indicated that low access of health service and treatment was one of the major consequences of violence, as reported by 31 (77.5%) participants. This finding is corroborated by previous qualitative study on HIV positive women in Nepal which identified terrible discrimination from family and community members (International Community of Women living with HIV/AIDS, 2009a). This study further investigated that HIV positive women were discriminated by even health personnel and this behaviour deterred them to seek health services and treatment. Likewise in India, 5 out of 7 HIV positive women willing to take free abortion services at government hospitals were denied because of the HIV status (International Community of Women living with HIV and AIDS, 2009b). In another study among HIV positive women in the UK, one-third of the participants felt that they were not accessing good GP care due to their HIV status (Petretti, 2009).

This study provides an important ‘snapshots’ on violence among HIV positive women in Nepal. However, the findings of this study should be interpreted with following caveats. Firstly, the participants of this study were selected by convenience sampling method rather than the random sampling method, hence we could not deny the possibility of selection bias. Secondly, there might be the possibility of re-call bias because it is possible that participants might have mostly remembered recent incidents of violence. Similarly, due to the limitations of data we could not explore social/demographic or HIV status related parameters to determine the factors for the occurrence of violence. Finally, this study was conducted on small sample size in three districts of Nepal, thus the findings should not be extrapolated to the country as a whole.

The results of this study have important implications for public health practice and further research. Legislation [Domestic Violence (Offence and Punishment) Act 2009] to minimize violence against women are already on place in Nepal, but effective implementation is still a huge challenge. Thus, we urge authorities to initiate actions in this regard with specially focus on HIV positive women. We also recommend to conduct advocacy and awareness campaign on violence against HIV positive women. Since the perpetrators are mostly the husband and close relatives, we recommend to extend and expedite family counselling intervention. HIV related stigma and discrimination should be addressed in policy as well, including at health care setting. In Nepal, HIV related programs are centred to reduce risky sex behaviour and prevention from HIV transmission. Now, it has been equally important to prioritize programs on social and behavioural aspects of HIV, for e.g. violence. Finally, further larger and nationally representative researches are imperative to better understand the cross-section between VAW and HIV.

## 5. Case Studies

i) Devi Chhetri (name changed), 34, is a HIV positive women from Bastipur, Makwanpur distict. At the age of 19, she fell in love with a truck driver and subsequently married with him. She gave birth to two daughters and one son. She heard promiscuous behaviour of her husband frequently but did not take it seriously.

In the year 2003, her husband was bed-ridden. None of the treatment was able to improve his health condition. Eventually, it was diagnosed that he was HIV positive. In the same year, he succumbed to the death.

After the death of sole breadwinner of the family, Devi was left in the lurch. She determined to check HIV status of herself and the children. Serum test result also showed HIV positive on her along with elder daughter. Fortunately, her one son and one daughter were safe from it.

Her family members began cruel behaviour after knowing her HIV status. They used to say that they did not have any family obligation and responsibility to take care since she had love marriage. Community also did not support her in any aspects. Gradually she was denied from basic needs and was compelled to leave the house. She had no option except working as a domestic worker.

Later, she joined Makwanpur Mahila Samuha (MMS), an organization working on HIV related issues. She was provided with the job of Peer Educator in MMS. Now, she is surviving an average life on her own capacity.

On her experience, HIV positive women cannot get support from the community or other people unless her own family is not supportive. Surprisingly, the family members who had forced her out of the home, are now behaving with good manner. “I can survive on my own and even able to save money, thus they might have changed the attitude towards me and my children”, she said.

ii) Binita Sharma (name changed), resident of Jutpani, Chitwan was married at the childhood age of 11. Her family was living in a dire poverty. For the sake of employment, her husband went to Mumbai, India. Her husband came back from India with serious illness. Primary treatment could not cure him. Finally, in the blood test, he was diagnosed HIV positive. Unfortunately, HIV positive was found in Binita’s blood test as well.

Her life-cycle took nasty turn after the death of her husband. She was treated with tremendous discrimination, harassment and hatred. She was ostracized by her family members and under the influence of other family members even her son refused to talk with her. She was denied to stay at home, instead she was forced to stay at the cowshed. Being informed about her condition, a social activist of the village met with her and noted her condition. A social activist inspired Binita on the brighter side of the life and tried to raise her motivation towards the life. Binita’s in-laws were also counselled about the rights guaranteed by the existing laws. After several attempts, her in-laws held back from perpetrating cruel behaviour. Instead, they started to treat her alike other family members. She was provided with livelihood skills training from Chitwan Sakriya Samuha, an organisation run by HIV women for the HIV women. Now, life has been better for her. She is living dignified and optimistic life with the family members.
